# Virtual screening of ultra-large chemical libraries identifies cell-permeable small-molecule inhibitors of a “non-druggable” target, STAT3 N-terminal domain

**DOI:** 10.3389/fonc.2023.1144153

**Published:** 2023-04-25

**Authors:** Pedro Andrade Bonilla, Cody L. Hoop, Karen Stefanisko, Sergey G. Tarasov, Sourav Sinha, Marc C. Nicklaus, Nadya I. Tarasova

**Affiliations:** ^1^ Cancer Innovation Laboratory, Center for Cancer Research, National Cancer Institute, National Institutes of Health, Frederick, MD, United States; ^2^ Center for Structural Biology, Center for Cancer Research, National Cancer Institute, National Institutes of Health, Frederick, MD, United States; ^3^ Oncolinx Inc., New York, NY, United States; ^4^ Computer-Aided Drug Design Group, Chemical Biology Laboratory, Center for Cancer Research, National Cancer Institute, National Institute of Health (NIH), Frederick, MD, United States

**Keywords:** virtual screening, transcription factor, chemical space, virtual libraries, microscale thermophoresis

## Abstract

STAT3 N-terminal domain is a promising molecular target for cancer treatment and modulation of immune responses. However, STAT3 is localized in the cytoplasm, mitochondria, and nuclei, and thus, is inaccessible to therapeutic antibodies. Its N-terminal domain lacks deep pockets on the surface and represents a typical “non-druggable” protein. In order to successfully identify potent and selective inhibitors of the domain, we have used virtual screening of billion structure-sized virtual libraries of make-on-demand screening samples. The results suggest that the expansion of accessible chemical space by cutting-edge ultra-large virtual compound databases can lead to successful development of small molecule drugs for hard-to-target intracellular proteins.

## Introduction

STAT3 has been long considered a promising drug target due to its involvement in proliferation of tumor cells, inflammation, and immune responses (1, 2). Being a transcription factor, it has however been widely considered a difficult target. Major efforts in the past have been directed towards indirect inhibition of the pathway by attenuating STAT3 phosphorylation *via* inhibition of up-stream kinases or phosphatases. However, indirect inhibitors suffer from low selectivity and, in several cases, low efficacy. For example, a Phase III clinical trial of the oral investigational agent napabucasin (also known as BBI608), which affects multiple oncogenic cellular pathways including STAT3 (3), has been recently discontinued due to futility in pancreatic cancer. Consequently, many research groups have continued with efforts to target the STAT3 protein directly (4). Like the other six members of the STAT family of transcription factors, STAT3 contains 6 domains (5). The N-terminal domain (ND) is frequently called “protein binding domain”. It is followed in the sequence by a coiled-coil domain, DNA-binding domain, linker domain (LD), Src Homology 2 (SH2) domain, and trans-activation domain (TAD). The Human Protein Reference Database (http://www.hprd.org/) lists 103 known direct binding partners of STAT3. The large number of protein-protein interactions reflects the complexity of STAT3 function and suggests that inhibitions of different domains is very likely to have significantly different functional consequences (6). Currently, there are three types of STAT3 inhibitors in clinical trials that target the protein directly: STAT3 antisense oligonucleotides, such as AZD9150 (7); small-molecule inhibitors targeting the STAT3 phosphotyrosyl peptide binding site within the SH2 domain, such as TTI-101 (8, 9) (https://clinicaltrials.gov/ct2/show/NCT03195699); and KT-333, a small molecule targeted protein degrader(https://clinicaltrials.gov/ct2/show/NCT05225584). Oligonucleotides suffer from difficulties in delivery and are currently administered by intra-tumoral injections only. STAT3 ND has emerged as a very different target (10). Inhibitors of this domain produce significantly different cellular and molecular effects compared to other STAT3 inhibitors (11, 12). The major functional difference is that they impact not only tumor cell proliferation, but also immune responses to tumors and bacterial pathogens. We have previously developed selective cell-permeable lipopeptide inhibitors of the domain (6, 11, 12). These inhibitors turned out to be a valuable tool in the characterization of the domain as a drug target. They have shown that inhibition of the domain induces expression of pro-apoptotic genes in cancerous, but not normal, cells (12). Administration of STAT3 ND peptide inhibitors to mice chronically infected with Mycobacterium tuberculosis resulted in clearing of the infection without the use of antibiotics, which was based on interference with IL10 signaling and reduction of the number of Treg cells in the lung (13).

Although peptides showed remarkable effects on both tumor cells and immune system cells, the problems with administration and short life in circulation prompted us to attempt the development of small molecule inhibitors. Small molecules have better ability to penetrate cellular membranes than peptides. However, they have a poor reputation in targeting non-druggable proteins, such as transcription factors, and protein-protein interactions. We hypothesized that their bad record in targeting such targets was partially due to the limited size and diversity of the compounds’ libraries commonly used in drug discovery. Consequently, expansion of accessible chemical space may open up the development of small molecule inhibitors for non-druggable intracellular oncoproteins thus addressing both poor druggability and drug accessibility. It has been shown that ultra-large library docking can help discover new chemotypes (14). A recent review discusses the exploration of ultra-large compound collections for drug discovery (15).

The X-ray structure of the STAT3 N-terminal domain (PDB 4ZIA) solved by a group from Novartis (10) has made virtual screens and structure-based design via, e.g., docking possible. Availability of new billion-sized libraries of synthetically accessible compounds including the Synthetically Accessible Virtual Inventory (SAVI) developed in this group (16), allowed for exploration of much expanded structural diversity that resulted in identification of potent and selective STAT3 N-domain inhibitors.

## Materials and methods

### Protein expression and purification

The plasmid containing the STAT3 2-124 sequence followed by 6 x His tag cloned into the bacteriophage T7 promoter vector pET3a was a kind gift from Jess Li (NCI). The plasmid was transformed into E. coli BL21(DE3) (Life Technologies). Single colonies were inoculated into 10 ml of LB broth supplemented with 50 mg/liter carbenicillin and grown at 37°C overnight. On the next day, the overnight culture was amplified into 1 liter of TB medium with 50 μg/ml carbenicillin. The culture was grown at 37°C for 3 to 4 h until the optical density at 600 nm reached 2.0 and induced with 0.4 mM isopropyl-beta-D-thiogalactoside (IPTG) at 17°C overnight. The cells were harvested by centrifugation at 6,000 g for 10 min at 4°C. The pellet was frozen and resuspended in 30 ml binding buffer (50 mM Tris-HCl pH 8.0, 500 mM NaCl, 1 mM tris(2-carboxyethyl) phosphine (TCEP), 10% glycerol) supplemented with complete protease inhibitors cocktail (Roche, Indianapolis, IN) and 0.1 mg/ml DNase I from bovine pancreas (Sigma) per 50 ml of lysis buffer. Cells were lysed by sonication with a large Hielscher’s probe for 35 seconds. The suspension was diluted 2 times with binding buffer and sonicated four more times for 35 sec with 1 sec intervals. The lysate was centrifuged for 45 min at 25000g and 4°C. Supernatant was loaded onto a5ml Ni-NTA column that was pre-equilibrated with lysis buffer. After washing with binding buffer plus 20 mM imidazole, the protein was eluted with binding buffer plus 300 mM imidazole. The eluted protein was applied to a HiLoad^®^ 16/600 Superdex^®^ 75 pg size exclusion column (Cytiva) equilibrated with 20 mM Tris-HCl pH 7.5, 150 mM NaCl, and 1 mM dithiothreitol (DTT). Peak fractions were analyzed using 6100 Series Single Quadrupole LC/MS. Experimental molecular mass was 15551.3 Da. Sequence correctness was further confirmed by analyzing trypsin digest of the protein on 6520 Accurate-Mass Quadrupole Time-of-Flight (Q-TOF) LC/MS/MS (Agilent Technologies, Inc.)

### Compounds

All compounds have been synthesized by Enamine Ltd (Kiev, Ukraine) and had purity of more than 95% according to LC/MS data.

### Microscale thermophoresis

For the microscale thermophoresis (MST) studies we prepared 16 two-fold serial dilutions of compounds starting from 100 μM. Titration series were prepared that contained 10 μL of 50 nM His-Tag RED-tris-NTA labeled STAT3 ND and 10 μL of compounds’ solutions of varying concentrations. Final buffer composition included 1X PBS containing 0.05% Tween-20 and 0.5% DMSO. All measurements were taken in standard treated capillaries on a Monolith NT.115 instrument (NanoTemper Technologies, Munich, Germany) using 50% infrared laser power and an LED excitation source with λ = 650 nm. NanoTemper Analysis 1.2.20 software was used to fit the data and to determine the KD values.

### Cell toxicity assay

DU145 cells were seeded in Eagle’s minimum essential medium containing 2% fetal bovine serum. PC-3 cells were seeded in F-12K medium containing 1% fetal bovine serum. MDA-MB-231 cells were seeded in DMEM medium and 2% fetal bovine serum final concentration. All cells were seeded at 2000 cells/well in 96-well plates. After 24 h, cells were treated with 0.5% DMSO (as a control) and this concentration was maintained with all compounds used at 4 µM, 2 µM, 1 µM, 0.5 µM or 0.25 µM final concentrations. Each condition was performed in sextuplicate. After 48 h, 0.35 mg/mL MTT was added for 4 h of incubation. Stop solution (40% DMF, 10% SDS (W/V), 25 mM HCl, 2.5% acetic acid in H_2_O) was added to the cells and incubated overnight. Absorbance at 570 nm was measured by using CLARIOstar (BMG LABTECH, Ortenberg, Germany).

### Western blot analysis

DU145 cells grown to 80% confluency were treated for 3 hours with the compounds (with DMSO treatment as negative control) and the cytoplasmic and nuclear fractions were isolated as described (12). STDND-2, STDND-9 and ST3H2-Pal9 were tested at 3µM and 10µM concentration. C-Fos rabbit monoclonal antibody (9F6, Cell Signaling Technology, Danvers, MA) was diluted 1:1000. The blot was stripped and re-probed with anti-β-actin mouse monoclonal antibodies (8H10D10, Cell Signaling Technology) diluted 1:1000.

### HEK-BLUE IL-10 reporter cells assay

180 µl of HEK-BLUE IL-10 reporter cells suspension containing 70000 cells/ml in DMEM supplemented with 4.5 g/l glucose, 2 mM L-glutamine, 10% heat inactivate FBS, Pen-Strep mixture (100 U/ml, 100 µg/ml) were added per well in 96-well plates. Then, cells were treated with 20 µl of compound solutions at four different concentrations 50 µM, 25 µM, 12.5 µM, and 6.25 µM in 5% DMSO.

After 24 hours of incubation, 20 µl of supernatant were aspirated from each well, transferred to a 96-well assay plate and incubated with 180 µl of Quanti-Blue secreted embryonic alkaline phosphatase (SEAP) substrate solution for 2 hours. The levels of SEAP were determined by measuring the absorbance at 640 nm using CLARIOstar (BMG LABTECH) plate reader.

### Ultra-large libraries used

We used two libraries: REAL (https://enamine.net/compound-collections/real-compounds/real-database), and SAVI, developed in-house (but publicly available) (16). Both databases are based on building blocks from Enamine (Kyiv, Ukraine). Because compounds from both REAL and SAVI can be directly ordered from Enamine, we typically screened both libraries together. At the time of this screening, REAL had about 1.2 billion compounds. SAVI (in its 2020 version) has 1.75 billion molecules; however, only the highest-synthesizability subset of SAVI was used for screening, consisting of about 1 billion structures. Even though REAL and SAVI are based on a significantly overlapping building block set, they are crucially different in several ways: (1) They use different chemical rules (“transforms”) for the creation of the virtual molecules and their predicted synthetic routes. (2) SAVI, in contrast to most other ultra-large libraries (which are by necessity virtual; see 15, for an overview), is based on an expert-system type approach that uses chemical programming language CHMTRN (CHeMistry TRaNslator), recently adapted for forward-synthetic application (17), providing a much more powerful description of chemical synthesis knowledge than most other approaches (such as SMIKRS-based rules), thereby yielding higher success rates of syntheses (3). The SAVI methodology enables one to quickly generate new transforms for novel chemistry without having to wait for large databases of reactions to emerge needed to do machine-learning analysis.

### In silico screening

Docking screens were conducted using the ICM-Pro (Molsoft L.C.C., San Diego, CA) software by running up to 1000 parallel processes on up to 6000 CPUs of the National Institutes of Health (NIH) Biowulf cluster supercomputer. The PoketFinder software (Molsoft) was used for identification of the pockets. Screens were run in large-scale parallel way as so-called “swarm” jobs. In initial fast screens, each job screened 1000-10000 compounds and used 1 CPU. Up to 1000 jobs were run in parallel for each screen. The hits from the initial screens were used in the secondary, much slower screens run with thorough factor of 100 and having 5 compounds assigned to each. 20–30 top compounds from the second round of screens were finally redocked manually, and the best-scoring compounds selected for ordering/synthesis and subsequent experimental testing.

### Plasma stability studies

Plasma stability studies were conducted by WuXi AppTec Co. Ltd. ((Nanjing, China). The concentration of ST3ND-25 and ST3ND34 in plasma was determined using SCIEX Triple Quad 6500+ LC-system and Waters Acquity UPLC BEH C18 1.7 μm 2.1 × 50 mm Column. 2 μl of 100 μM compound stock solution in DMSO were added to 98 μl of thawed pooled plasma that was cleared by centrifugation for 5 min at 4000 rpm. The mixtures were incubated at 37°C and precipitated with 500 μl of acetonitrile containing internal standards, 200 ng/ml of tolbutamide and 200 ng/ml of labetalol. After 20 min of mixing, the 96-well plate with samples was centrifuged for 20 min at 4000 rpm and supernatants were analyzed by LC-MS/MS.

## Results

**Figure 1:**
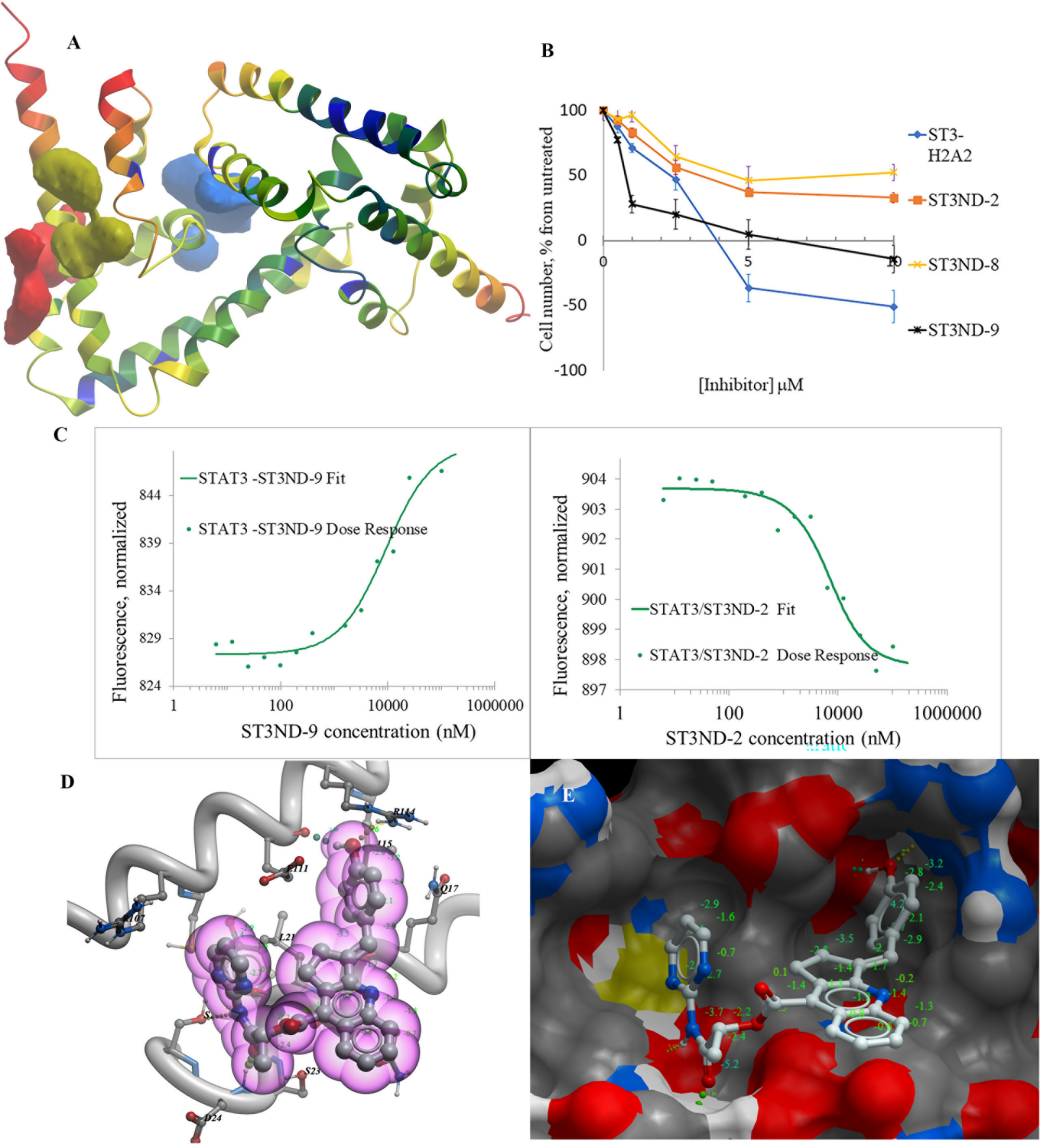
Structure-based development of STAT3 N-domain inhibitors: **(A)**, Structure of STAT3 N-domain dimer (PDB 4ZIA). The best binding pocket is in green. The next most druggable binding pockets are shown in blue and red, respectively. Protein ribbon is colored by B-factor with red representing the most mobile parts, and blue corresponding to the best-resolved ones. All pockets reside in mobile areas of the structure; **(B)**, An example of STAT3 N-domain inhibitors effects on the growth of prostate cancer DU145 cells. Cells have been exposed to compounds for 48 hours and the number of surviving cells was quantitated using MTT assay. N-terminal domain peptide inhibitor ST3H2-A2 (12) was used as a positive control; **(C)** The examples of Microscale Thermophoresis (MST) binding assays- titration of hit compound, ST3ND-9, into recombinant STAT3 N-terminal domain. His-tagged STAT3 N-domain was labeled with non-covalent fluorescent dye RED-tris-NTA for detection by MST. The protein concentration was 50 nM. Apparent K_d_=7.3 μM; **(D)**, STND-9 compound docked into STAT3 N-terminal domain structure. Phenol group of the hit fits well in the pocket. **(E)**, STND-9’s interactions with protein’s side groups and the main chain.

Since the resolution of the available crystal structure was only 2.7Å, we ran extensive minimization of the side chains using ICM-Pro software (Molsoft, L.L.C.) prior to using the structure in the screens. PocketFinder software from Molsoft identified the three best pockets on the protein’s surface (**Figure 1A**). The druggability of all three was predicted to be poor with drug-like density (DLID) (18) of -0.37 for the best pocket depicted in green in **Figure 1A** and -0.57 and -0.66 for the blue and red ones, respectively. Diagnostic screens against Enamine’s database of in-stock compounds did not produce high-scoring hits for the latter two pockets but allowed for identification of several hits for the green one. We focused our screening efforts on this pocket not only because it had the highest DLID, but also because it was formed by helix2 of the protein, which was used in the past for the generation of dominant-negative inhibitors of the domain (11, 12).

For virtual screening, we used ICM-Pro software in VLS mode, run on the NIH Biowulf supercomputer cluster. ICM-Pro from MolSoft (San Diego, CA), based on Internal Coordinate Mechanics (ICM) (19), was selected because it had ranked at the top in several benchmarks in recent years (20–22). The Enamine library of in-stock compounds containing almost 2 million entries was used for initial screens, which evaluated the pockets and identified early hits (STND1-7, **Table 1**). Initial fast screen with the docking parameter value thorough=1 produced 1335 potential hits for the green pocket. More thorough docking of these hits with subsequent manual redocking of the top 25 hits yielded 7 best-scoring compounds, which were ordered for experimental analysis. Binding of the compounds to purified His-tagged STAT3 N-domain labeled with non-covalent fluorescent dye RED-tris-NTA was assessed by Microscale Thermophoresis (MST). It should be noted that MST does not always detect binding of small molecules to a protein because the changes in protein-solvent interaction can be too small to impact protein diffusion in a gradient of temperature. Nevertheless, 5 out of 7 compounds produced detectable change in the MST signal with K_D_s between 7 and 179 μM (**Table 1**, **Figure 1C**), thus proving the direct interaction with the protein. The biological activity of the compounds was tested by toxicity assay on three STAT3 N-domain-dependent cell lines, breast cancer MDA-MB-231, and prostate cancer PC-3 and DU145. ST3ND-1, 2 and 5 were toxic to STAT3 ND dependent cells with GI50 values in the low micromolar range (**Table 1**, **Figure 1B**). Encouraged by the accuracy of these initial virtual screen predictions, we expanded docking screens to two larger Enamine diversity libraries containing 5 and 15 million compounds, respectively, and to our in-house developed library, SAVI. Screening the entire REAL or SAVI libraries by docking with ICM-Pro is impractical as it would take around 14 million CPU hours, i.e., the better part of a year, even with the maximal resources available per user group on NIH supercomputer cluster Biowulf. For SAVI, we therefore generated a diversity set containing 2,955,416 entries. Any combination of compounds in the set has a Tanimoto similarity index score of T < 0.6, making the library a valuable tool for identification of diverse hits for optimization. However, compounds in diversity sets, like REAL and the rest of SAVI, need to be typically synthesized, since the vast majority are of these compounds are not commercially available off-the-shelf and in fact have never been synthesized before. These syntheses are however straightforward in most cases, as all of the SAVI compounds are predicted to be synthesizable in one-step syntheses from Enamine building blocks, and most REAL compounds are based on one- to three-step syntheses. The best hits from screening these diversity sets, STND-11-20 (**Table 2**) were synthesized by Enamine and tested for STAT3-dependent tumor cell grows inhibition.

**Table 1:** Compounds selected for testing after docking of Enamine’s screening compounds database.

Compound	Structure	MST K_d_ (μM)	GI_50_, MBA- MB-231, μM	GI_50_, PC-3, μM	GI_50_, DU145, μM
ST3ND-1	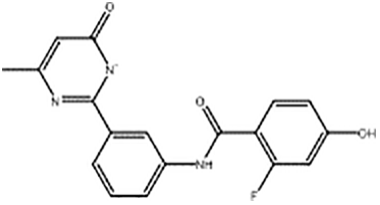	23.1 ± 7.9	4	7	>10
ST3ND-2	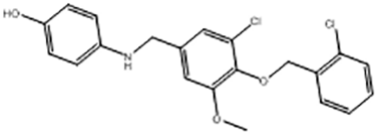	7.5 ± 2.3	3.5	>10	0.8
ST3ND-3	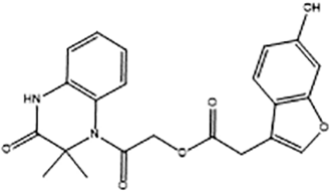	179 ± 22	>10	>10	>10
ST3ND-4	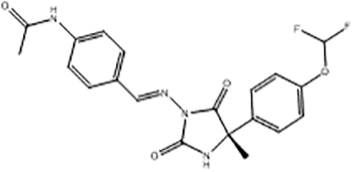	8.6 ± 1.5	>10	>10	>10
ST3ND-5	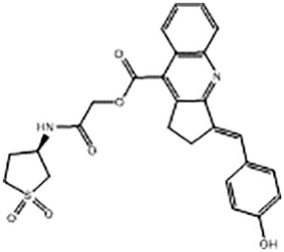	> 200	3	10	>10
ST3ND-6	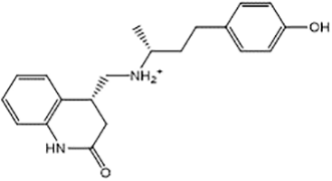	> 200	>10	>10	>10
ST3ND-7	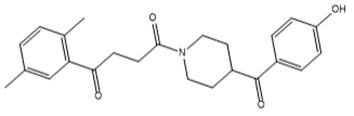	68 ± 36	>10	>10	>10

Toxicity was evaluated for three STAT3 N-domain dependent cancer cell lines.

Binding constants were determined by Microscale Thermophoresis (MST) of recombinant fluorescently labeled STAT3 N-terminal domain.

**Table 2:** Compounds selected for testing after docking of REAL and SAVI diversity sets and subset of subsets of whole REAL and SAVI databases identified using similarity searches.

Name	Structure	IC_50_, DU145, μM
ST3ND-8	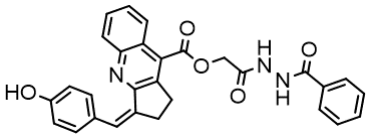	0.8
ST3ND-9	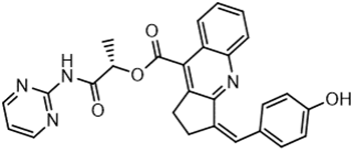	0.5
ST3ND-10	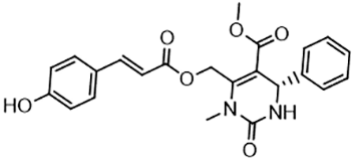	9
ST3ND-11	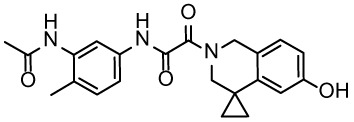	>10
ST3ND-12	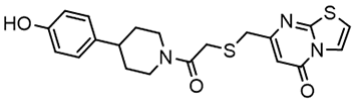	>10
ST3ND-13	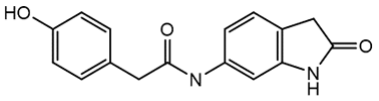	>10
ST3ND-14	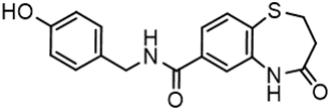	>10
ST3ND-15	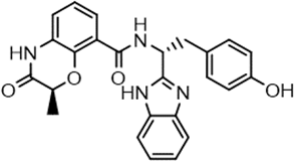	>10
ST3ND-16	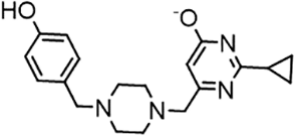	>10
ST3ND-17	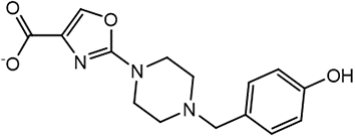	>10
ST3ND-18	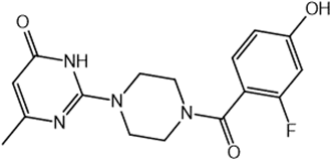	>10
ST3ND-19	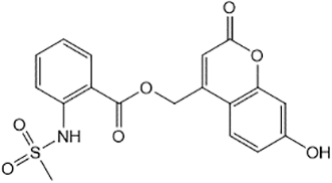	>10
ST3ND-20	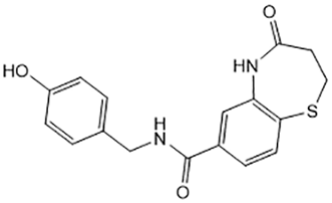	>10
ST3ND-21	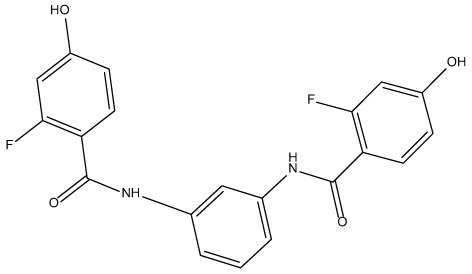	>4
ST3ND-22	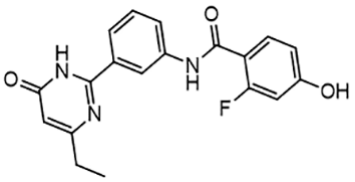	>4
ST3ND-23	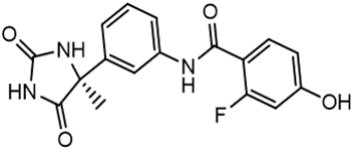	>4
ST3ND-24	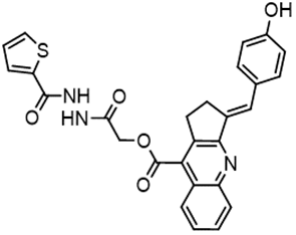	3.8
ST3ND-25	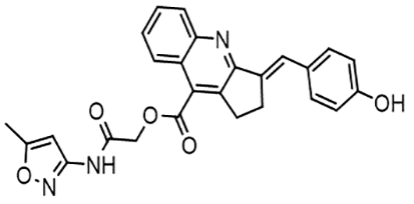	0.5
ST3ND-26	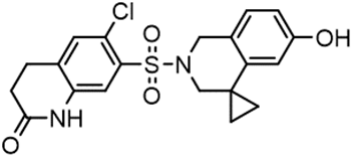	>4
ST3ND-27	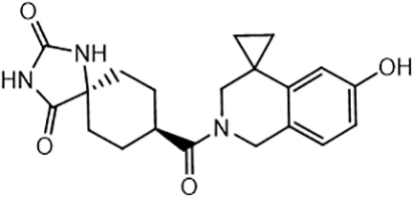	>4
ST3ND-28	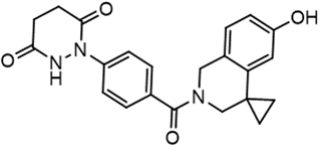	>4
ST3ND-29	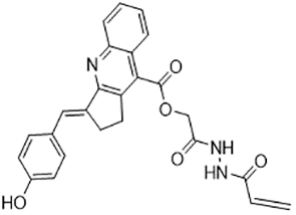	>4
ST3ND-30	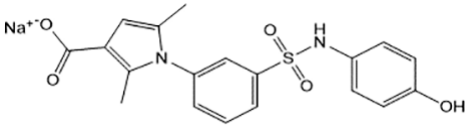	2
ST3ND-31	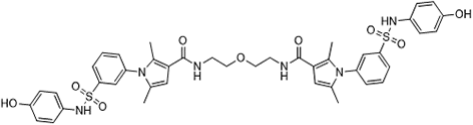	> 4
ST3ND-32	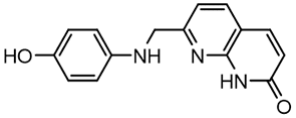	> 4
ST3ND-33	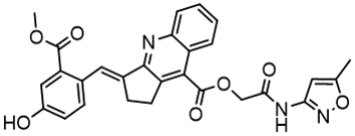	5
ST3ND-34	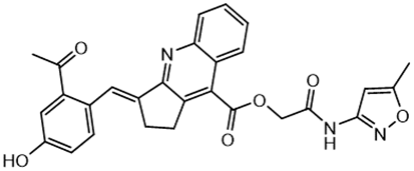	0.4
ST3ND-35	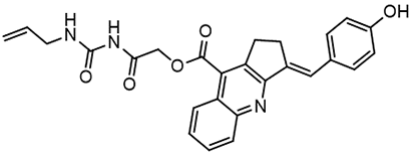	1.3
ST3ND-36	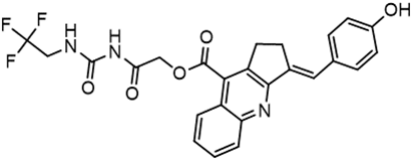	1
ST3ND-37	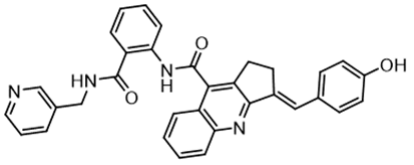	1

Biological activity was evaluated using STAT3 N-terminal domain dependent prostate cancer DU145cell line.

In parallel, the scaffolds producing the most active compounds, ST3ND-2 and ST3ND-5, were screened in similarity searches to identify potential binders in similar chemical structures. The 3D similarity search engine GIGA (https://www.molsoft.com/giga-search.html) was used to screen the REAL database of 1.2 billion synthesizable compounds from Enamine (https://enamine.net/library-synthesis/real-compounds/real-database). The web tool SciWalker (OntoChem, https://ontochem.com/2020/11/05/sciwalker-and-sciwalker-studio-as-integrated-tools-for-knowledge-extraction/) was used for searching the SAVI database. The sets of potential hits identified by these similarity searches were subjected to the three-step docking screen procedure described above that has proven to reliably identify effective binders. Activity testing allowed for validation of virtual screen results (compounds ST3ND-8-10, 21-28 and 35-36) and provided insights into structure-activity relationships within the series. Screening large diverse libraries thus permits “virtual medicinal chemistry,” allowing to identify structural motifs critical for the activity. As can be noted from **Tables 1** and **2**, most hits contain a phenolic group that appears to contribute significantly to the binding. It fits well in the pocket, and forms hydrogen bonds with the amide carbonyl of Glu111 and the guanido group of Arg114 (**Figure 1D**), thus adding significantly to interaction energy. As the most active compounds have been derived from (Z)-3-(4-hydroxybenzylidene)-2,3-dihydro-1H-cyclopenta[b]quinoline-9-carboxylic acid, we proceeded with optimization of these derivatives using Molsoft’s ligand editor. The best-scoring compounds, ST3ND- 33 and 34, were synthesized and assessed for binding and biological activity. Only compound 34 showed some improvement in activity compared to the best compound identified from the screens, ST3ND-25.

**Figure 2:**
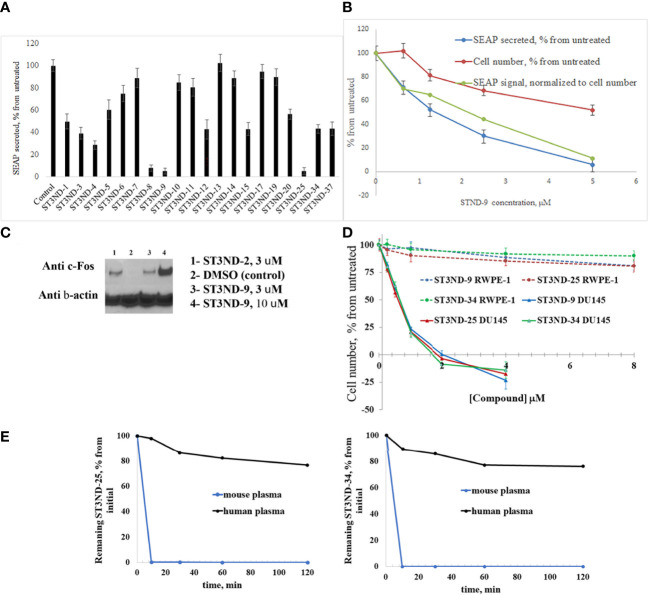
Compounds interfere with STAT3 N-domain signaling. **(A)**, STAT3 ND binders inhibit STAT3 signaling in HEK-BLUE IL-10 reporter cells. Cells (~70,000 cells/mL) were incubated with IL-10 (0.1 ng/ml) without or with 500μM of compound by measuring secreted embryonic alkaline phosphatase (SEAP) levels induced by 24 hours incubation). **(B)**. Inhibition of IL-10 induced STAT3 signaling in HEK-BLUE IL-10 reporter cells was concentration-dependent. As expected, compounds exhibited cell toxicity (blue line). However, inhibition of STAT3 activity was more significant and did not appear to be toxicity mediated. **(C)**, Compounds induce expression of STAT3 ND inhibition marker c-Fos. DU145 cells were treated with compounds for 3 hours (with DMSO treatment as a negative control), and nuclear fraction was isolated and used for Western blot analysis as described(12). Anti-c-Fos antibody was used at 1:1000 dilution. The blot was stripped and re-probed with β-Actin antibody at 1:1000 to demonstrate equal loading of the wells. **(D)**. Compounds are selectively toxic to prostate tumor cells (DU145) and show very little toxicity to normal prostate epithelial cells (RWPE-1). **(E)**, Lead compounds ST3ND-25 and ST3ND-34 are stable in human plasma, but undergo rapid metabolism in mouse plasma.

To evaluate the effects of compounds on STAT3 signaling, we tested their inhibition of Il-10- induced secretion of embryonic alkaline phosphatase in reporter cell line, HEK-Blue IL10 cells (Invivogen, https://www.invivogen.com/hek-blue-il10). Compounds not only effectively interfered with STAT3 activity, but the degree of inhibition by different compounds correlated well with the binding score and toxicity in STAT3-dependent cancer cells (**Figure 2A**). The effects on STAT3 signaling were concentration dependent (**Figure 2B**). As expected, compounds did show some toxicity on HEK cells. However, it was less pronounced than effects on the signaling, strongly suggested that the observed reduction in transcription of the marker gene was not a result of cells loss.

Peptide inhibitors of STAT3 ND have been shown to up-regulate expression of several proapoptotic proteins, in particular c-Fos (12). Western blot analysis of c-Fos expression showed that the small-molecule domain inhibitors also effectively increase c-Fos expression in prostate cancer cells (**Figure 2C**). Compounds were selectively toxic to prostate tumor cells (DU145) and showed very little toxicity to normal prostate epithelial cells (RWPE-1) (**Figure 2D**).

Before proceeding to testing the compounds in animal models, we tested stability of two lead compounds, ST3ND-25 and ST3ND-34 in human and mouse plasma. While the compounds were stable in human plasma with half-lives beyond 290 min, they unexpectedly appeared to be rapidly metabolized in mouse plasma, showing half-lives of less than 10 min (**Figure 2E**). Mice, unlike humans, have high levels of esterase activity in plasma (23). It is a known concern in using mouse preclinical models (24) that can impede further preclinical evaluation of compounds. Both compounds have an ester bond in their structures. Although the bond is formed by a bulky acid residue that tends to provide for better stability, hydrolysis by plasma esterases may still be possible.

## Discussion

Although the STAT3 N-domain has all the traits of a very challenging drug target, screening of large virtual libraries allowed for identification of small molecule inhibitors that are more than an order of magnitude more potent than previously described and used peptide inhibitors. They show very similar *in vitro* effects on STAT3 signaling, and consequently can be used for both tumor growth inhibition and modulation of immune responses to tumors and infectious agents.

The identified leads ST3ND-25 and ST3ND-34 are unstable in mouse plasma, despite being stable in human plasma. Hydrolysis of an ester bond by a plasma esterase is the most likely route of mouse plasma metabolism. Because of instability in mouse plasma, preclinical development of compounds with this scaffold will be challenging. The structures, however, can be used in screening of mega-libraries of compounds using 3D-similarity searches that allow for scaffold hopping. One such approach, Rapid Isostere Discovery Engine (RIDE, https://www.molsoft.com/RIDE.html) has been recently developed by Molsoft. It is based on Atomic Property Fields (APF) (25, 26) that represent 3D pharmacophore potential, which can be used for chemical superposition, screening, and scaffold hopping. Current work has shown that billion-sized libraries of compounds are a promising source of ligands for non-druggable protein targets, and that STAT3 ND can be targeted with small molecules.

Further work in this area is warranted because compounds can have many clinical applications in addition to cancer therapy: Effects of STAT3 ablation in natural killer (NK) cells suggested that STAT3 inhibitors can be used to stimulate cytolytic activity of NK cells against leukemia (27). In addition, clinical trials of non-genetically modified NK cells show promising results both in hematological malignancies and solid tumors (28). However, methods of ex-vivo activation of NK cells are still in development. Thus, STAT3 ND inhibitors can be used for NK cell activation and prevention of NK cell exhaustion ex-vivo and *in vivo*.

Similarly, STAT3 has been shown to inhibit expression of cytotoxic genes in CD8+ T cells (29). The ability to manipulate and target this pathway with STAT3 ND inhibitors might be a valuable approach to enhance antitumor responses in cancer immunotherapy strategies.

We have shown previously that STAT3 ND peptide inhibitors can clear the lungs of mice chronically infected with Mycobacterium tuberculosis without the use of antibiotics due to activation of immune responses to the bacteria (13). Such inhibitors can potentially facilitate treatment of many pathological conditions caused by viruses and bacteria and could be used as adjuvants for vaccines.

Aberrant STAT3 pathway has been shown to play a critical role in the pathogenesis of SARS-CoV-2 (30). STAT3 inhibition was suggested as a treatment strategy for COVID-19 symptoms. Thus, STAT3 ND inhibitors may be useful for therapy of COVID-19.

## Data availability statement

The original contributions presented in the study are included in the article. Further inquiries can be directed to the corresponding author.

## Author contributions

PB conducted binding and activity assays, analyzed the data, and wrote the manuscript. CH conducted virtual screens, binding and activity assays, analyzed the data, and wrote the manuscript. KS expressed and purified the protein, conducted biological activity assays, and analyzed the data. ST designed the binding assays, analyzed the data, and wrote the manuscript. SS designed activity assays and analyzed the data. MN designed and generated virtual libraries, designed ligand-based virtual screens, analyzed the data, and wrote the manuscript. NT designed the study, conducted virtual screens, analyzed the data, and wrote the manuscript. All authors contributed to the article and approved the submitted version.
